# Antifungal Activity of Citrus Essential Oil in Controlling Sour Rot in Tahiti Acid Lime Fruits

**DOI:** 10.3390/plants13213075

**Published:** 2024-11-01

**Authors:** Vanessa Santos Moura, Lara Dias Olandin, Beatriz Saraiva Mariano, Josiane Rodrigues, Fernando Trevizan Devite, Ana Carolina Costa Arantes, Carmen Lucia Queiroga, Adilson Sartoratto, Fernando Alves de Azevedo, Marinês Bastianel

**Affiliations:** 1 Centro de Citricultura Sylvio Moreira, Instituto Agronômico, Cordeirópolis 13492-442, SP, Brazil; vanessa_smoura@hotmail.com (V.S.M.); laraorlandini@outlook.com (L.D.O.); beatriz.saraiva@estudante.ufscar.br (B.S.M.); devite_fernando@hotmail.com (F.T.D.); accarantes@gmail.com (A.C.C.A.); mbastianel@ccsm.br (M.B.); 2 Centro de Ciências Agrárias, Universidade Federal de São Carlos, Araras 13600-970, SP, Brazil; josirodrigues@ufscar.br; 3 Centro Pluridisciplinar de Pesquisas Químicas, Biológicas e Agrícolas, Universidade Estadual de Campinas, Campinas 13148-218, SP, Brazil; queiroga@unicamp.br (C.L.Q.); adilson@cpqba.unicamp.br (A.S.)

**Keywords:** natural fungicides, post-harvest, biocontrol, agricultural sustainability

## Abstract

Sour rot, caused by *Geotrichum citri-aurantii*, is a significant post-harvest disease in citrus, resulting in economic losses due to the lack of effective fungicides. This study investigates the antifungal activity of citrus essential oils in controlling sour rot in Tahiti acid lime fruits. Essential oils were extracted via hydrodistillation with chemical composition analyzed by CG-MS and tested in vitro and in vivo. In vitro assays evaluated mycelial growth inhibition at 2 to 32 µL mL^−1^ concentrations. In vivo trials involved preventive and curative treatments on artificially inoculated fruits stored at 25 °C ± 2, and the results showed that Pera IAC sweet orange oil, at 32 µL mL^−1^, reduced disease severity by 96% in curative treatments. In contrast, Late IAC 585 willowleaf mandarin oil demonstrated moderate inhibition (44%) at the highest concentration in vitro. The oils did not affect key fruit quality parameters such as juice yield and total soluble solids. These findings suggest that citrus essential oils could be natural alternatives to synthetic fungicides for post-harvest sour rot management, combining effectiveness with maintaining fruit quality.

## 1. Introduction

Citrus farming plays a crucial role in global agriculture, with Brazil standing out as one of the leading citrus producers. In 2022, Brazil ranked as the fourth-largest global producer of limes and lemons, with an estimated production of 1.06 million tons [1]. Brazilian production is significant for the domestic fresh fruit market and a major exporter of frozen concentrated orange juice in volume and value [2]. Brazil exported, in 2023, about 167 thousand tons of Tahiti acid lime, mainly to Europe, representing an increase of 113% in the last 10 years [3]. However, India leads the global market, producing 3.77 million tons of limes and lemons in the same year [1].

In citrus, post-harvest diseases are a significant factor that reduces the quantity of fruit available to consumers, resulting in a decline in fruit quality and quantity from harvest to the point of sale [4]. Sour rot and green and blue molds, caused by *Geotrichum citri-aurantii*, *Penicillium digitatum*, and *Penicillium italicum*, are the main infections affecting citrus fruit post-harvest, leading to losses ranging from 25% to 50% of total production [5,6].

Sour rot is one of the most prevalent citrus diseases globally, affecting all cultivated varieties, including Tahiti acid lime, and primarily infecting fruit through wounds. Therefore, careful harvesting, handling, and storage are crucial to reducing the incidence of this disease. Many chemical fungicides used in post-harvest treatments, such as Thiabendazole and Imazalil, commonly used to control other post-harvest diseases (green or blue mold), are ineffective against *G. citri-aurantii* [7]. Moreover, some of these products face restrictions in certain countries, contribute to water and soil pollution, and promote increased pathogen resistance [8]. As a result, it is essential to find new, natural, safe, and environmentally friendly compounds to control sour rot during the post-harvest phase.

Studies indicate that essential oils extracted from plants act as natural fungicides, inhibiting fungal growth without leaving harmful toxic residues [9,10]. These oils work directly by inhibiting mycelial growth and spore germination and stimulating the production of phytoalexins in plants, acting as elicitors [11,12].

Essential oils can be extracted from plants through steam distillation, the most common method, and by cold pressing the peel of citrus fruits. These oils have perfumery, cosmetics, and food applications and are also used as herbicides, insecticides, and fungicides [13]. The compounds in essential oils can act directly on pathogens, altering the transmembrane potential, reducing ATP synthesis, and causing significant damage to DNA and mitochondria [14,15]

Due to the lack of effective commercial fungicides for controlling *G. citri-aurantii* [7], citrus essential oils are expected to replace traditional fungicides in managing post-harvest diseases due to their excellent multifaceted properties. In this study, we investigated the inhibitory efficacy of citrus essential oils against *G. citri-aurantii* in vitro, their effectiveness in controlling sour rot in vivo in Tahiti acid lime fruits, and their impact on fruit quality.

## 2. Results

### 2.1. Yield of Essential Oils (Eos)

The essential oil extraction yield from each variety investigated Late IAC 585, BRS Rainha, and Rio IAC 194 willowleaf mandarins (*Citrus* × *deliciosa*), Murcott IAC 221 tangor (*C.* × *sinensis* × *C. reticulata*), and Pera IAC sweet orange (*Citrus* × *sinensis*) and was quantified and expressed as a percentage according to Equation (1). The sample with the highest extraction yield was Late IAC 585 (0.16 mL 100 g^−1^), followed by BRS Rainha (0.13 mL 100 g^−1^) and Pera IAC (0.11 mL 100 g^−1^). Murcott IAC 221 tangor and Rio IAC 194 willowleaf had lower extraction yields, with 0.08 and 0.06 mL 100 g^−1^, respectively.

### 2.2. Chemical Composition of EOs by GC-MS

The chemical compositions of the EOs were determined using GC-FID and GC-MS chromatographic analyses, in which 38 volatile compounds were identified and 1 unidentified (Table 1), highlighting monoterpenes, sesquiterpenes, aldehydes, and alcohols, among others. The highest number of compounds were found in the Pera IAC oil (25 compounds) and BRS Rainha (28 compounds); on the other hand, a smaller number was observed in the Murcott IAC 221 essential oil (20 compounds), while intermediate values of components are present in the Late IAC 585 and Rio IAC 194 oils (23 compounds).

### 2.3. Bioactivity of Essential Oils on the Mycelial Growth of G. citri-aurantii

The Late IAC 585 and Pera IAC oils at a concentration of 32 µL mL^−1^ provided average inhibition rates of mycelial growth of *G. citri-aurantii* by direct contact of approximately 44% and 25%, respectively (Table 2).

Additionally, the oil from willowleaf mandarin (32 µL mL^−1^) resulted in a reduction of approximately 57% (MGRI) in the speed of mycelial growth of the pathogen compared to the control (Table 2). These results suggest that essential oils of both varieties inhibit *G. citri-aurantii* growth in a dose-dependent manner in vitro, making both samples viable for in vivo trials for disease control in fruit. Although the other oil samples, Rainha, Murcott, and Rio, reduced the growth rate index compared to the control and showed slight inhibition on the plates, they were less effective at inhibiting the pathogen’s mycelial growth.

**Figure 1:**
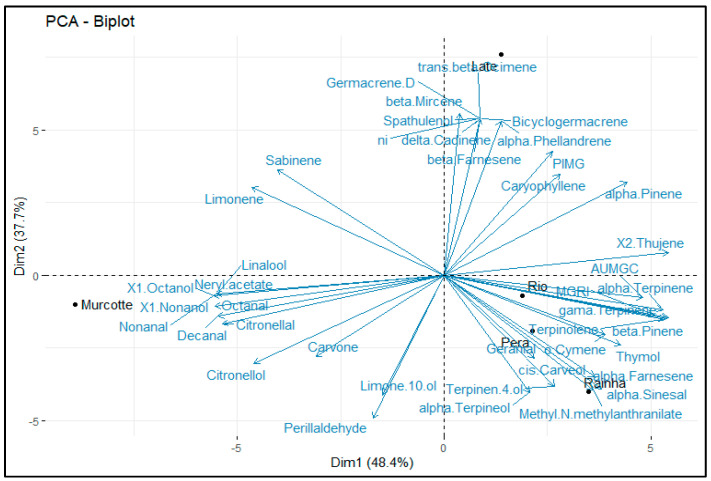
Biplot of 39 chemical components of the essential oils used (Late IAC 585, BRS Rainha, Rio IAC 194 willowleaf mandarins, Murcott IAC 221 tangor and Pera IAC sweet orange and MGRI, AUNGC, and PIMG values at a dose 32 µL mL.

Using the Biplot, it was possible to identify which compounds discriminated the samples the most and highlight the relationships between them (Figure 1). The first two components, CP1 and CP2, explained 86.1% of the variability between the descriptors, with the first component capturing 48.4% of the total data variability and the second 37.7% of this variation.

In general, it is possible to identify the presence of three segregated groups. The first group, formed by the Late sample, is represented almost entirely by the substances β-Mircine, Bicyclogermacrene, α-Phellandrene, and β-Farnesene. The second group, formed by the Rio, Pera, and Rainha samples, corresponds to the compounds γ-Terpinene, Methyl N-methyllanthranilate, α-Terpineol, β-Pinene, Terpinolene, Terpinen-4-ol, o-Cymene, and α-Terpinene. Finally, the third group is formed by Murcott tangor, with its compounds grouped as Linalool, Octanal, 1-Octanol, Citronellol, Decanal, Citronellal, and Nonanal.

Using the Biplot, it was also possible to identify that the PIMG values at a dose of 32 µL mL^−1^ were grouped together in the Late mandarin oil sample, while the AUMGC and MGRI values at the same dose were grouped together in the essential oil samples from the Rio, Pera, and Rainha varieties.

For the mycelial growth rate index, in the evaluation of antifungal activity, none of the oils evaluated showed a statistically significant difference from the control (Table 3).

However, regarding the AUMGC, it was noted that Late willowleaf mandarin and Pera IAC sweet orange oils, at doses of 16 and 32 µL mL^−1^, significantly differed from the control, with a mycelial inhibition percentage of *G. citri-aurantii* of approximately 31% for both doses. Rainha mandarin oil showed an inhibition percentage of around 25.7% compared to the control treatment, with no significant differences among the evaluated dosages. The other tested oils did not inhibit the pathogen’s mycelial growth.

### 2.4. Effectiveness of Essential Oils in Controlling Sour Rot in Post-Harvest Citrus

The results for disease severity showed that the contrast between the additional treatment (Control) and the triple factorial (Treatment × Oil × Dose) was not significant (*p*-value = 0.298), indicating that the means of the two groups are statistically equal. The interaction was insignificant when analyzing the triple factorial (Treatment × Oil × Dose) (*p*-value = 0.934). In this case, the breakdown of factors was examined: Treatment × Oils, Treatment × Doses, and Oils × Dose, where only the interactions Treatment × Doses and Oils × Dose were significant (*p*-value = 0.011 and *p*-value = 0.0068, respectively), as shown in Table 4 and Table 5. The data suggest that fruits treated curatively with Pera IAC sweet orange oil at a concentration of 32 µL mL^−1^ statistically differed from the other treatments, showing lower average lesion diameters of sour rot in Tahiti acid lime fruits stored at 25 °C ± 2.

Looking at the data in the three-fold split, it can also be inferred that in the preventive treatment with Late and Pera IAC oils, the 64 µL mL^−1^ dose resulted in a higher AUDPC compared to the 32 µL mL^−1^ dose, leading to a 48.84% reduction in average lesion diameter compared to the lower dose. In evaluating preventive and curative treatments at doses of 32 and 64 µL mL^−1^, there were no statistically significant differences compared to the control group for the average lesion diameter of sour rot. In the curative treatment, it was found that Pera IAC mandarin oil at 32 µL mL^−1^ resulted in a lower AUDPC than the higher dose, reducing disease severity by 99.3% (Supplementary Material).

Regarding the incidence of sour rot, there was no significant difference between the additional treatment and the triple factorial (*p*-value = 0.2178). The triple interaction of factors (Treatment × Oil × Dose) was insignificant (*p*-value = 0.2231). However, the two-way interactions Treatment × Dose (*p*-value = 1 × 10^−4^) and Oil x Dose (*p*-value = 0.0023) were significant (Table 6 and Table 7). Notably, fruits treated curatively with orange oil at 32 µL mL^−1^ showed a positive effect in controlling sour rot.

**Figure 2:**
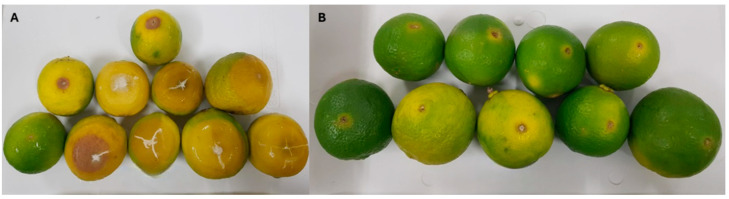
Tahiti acid lime fruits inoculated with *Geotrichum citri-aurantii* and curatively treated: (**A**) control (distilled water) and (**B**) Pera IAC sweet orange essential oil 32 µL mL^−1^. They were stored for 14 days at 25 °C ± 2 and 70% RH.

Tahiti acid lime fruits were preventively treated with Late oil at a concentration of 64 µL mL^−1^, and despite reducing the percentage of diseased fruits by 31.7%, there was no statistical difference from the control. We found that Tahiti acid lime fruits curatively treated with Pera IAC sweet orange oil at a concentration of 32 µL mL^−1^ showed a control efficiency of approximately 96% of sour rot about the control when we observed the three-way split of the data (Figure 2).

### 2.5. Fruit Quality in Citrus After Oleo Essential Treatment

The application of Late willowleaf mandarin and Pera IAC sweet orange essential oils at concentrations of 32 and 64 µL mL^−1^ did not significantly change the fruit quality parameters (average juice yield, total soluble solids (SS), total titratable acidity (TTA), and SS/TTA ratio) under commercial conditions when compared to the reference values determined by Pio et al. [16]. This indicates that the treated Tahiti acid lime fruits have commercial potential after applying the treatments (Table 8).

## 3. Discussion

Essential oils from Late IAC 585 willowleaf mandarin and Pera IAC sweet orange exhibited a slight inhibitory effect on mycelial growth in vitro using the agar diffusion method and volatile compound exposure assay, as well as a reduction in *G. citri-aurantii* sporulation, a pathogen that causes sour rot disease in citrus, one of the most challenging post-harvest diseases in citrus, leading to significant economic losses, exacerbated by the lack of effective fungicides [17]. The increasing resistance of fungi to synthetic fungicides, such as propiconazole and thiabendazole, necessitates alternative strategies that are both sustainable and environmentally safe [18]. In this context, essential oils have gained attention due to their antifungal properties and broad acceptance for controlling agricultural pathogens.

Gas chromatography coupled with mass spectrometry determined the composition of the EOs under study (Table 1). The major compounds identified were limonene (62.40–76.53%) and γ-terpinene (10.64–17.67%, except for Murcott tangor, 0.12%). Unfortunately, data on the chemical composition of the EOs alone are insufficient to associate the identified compounds with the observed biological activity.

Data from the literature demonstrate that some volatile compounds tested have activity against *G. citri-aurantii* and that, although they are present in some essential oils, their activity is concentration-dependent. Regnier et al. [19] tested 15 EOs and pure compounds and observed, for example, that citral (a mixture of geranial and neral) showed 75.6% of mycelial growth inhibition of *G. citri-aurantii*; however, *Cymbopogon citratus* (74.2% citral), *Litsea citrata* (72.9% citral), and *Lippia citriodora* (49.7% citral) showed 52.2, 56.4, and 64.0% of inhibition, respectively.

Zhou et al. [20] tested the antifungal activity of citral, octanal, and α-terpineol against *G. citri-aurantii.* They concluded that they can significantly inhibit the mycelial growth inhibition of *G. citri-aurantii*. The literature shows that essential oils [19], pure monoterpenes [20], and monoterpene mixtures (cinnamaldehyde and citral, 1:2, vv) [21] have the ability to inhibit the mycelial growth of *G. citri-aurantii*.

Our study and data from the literature [19] showed that limonene presented low inhibition (29.3%) of the mycelial growth of *G. citri-aurantii* and as it is present in high concentration in the studied samples with the best activity, Pera (*Citrus sinensis*) and Late (*C. deliciosa*), the results obtained here corroborate the limitation of associating the results of mycelial growth inhibition of *G. citri-aurantii* to the individual components present in the essential oils. However, it is important to present the chemical composition of the EOs so they can be used as a fingerprint and for future artificial intelligence studies.

Several studies have shown the effects of essential oils on the mycelial growth of phytopathogens. For example, mint oil inhibited *P. digitatum* and *G. citri-aurantii* for one week [22]. Essential oils from *Mentha piperita*, *Mentha spicata*, and *Mentha suaveolens* completely or nearly inhibited *Botryotinia fuckeliana* at 400 µg mL^−1^, with 92–100% mycelial growth inhibition. The same authors noted that reducing the dose of *M. suaveolens* oil to 200 µg mL^−1^ resulted in significantly lower inhibition levels [23]. Volatile citral applied at 60 mL L^−1^ showed potential for controlling sour rot; however, high concentrations of volatile citral may cause phytotoxicity symptoms in fruits [24]. According to Bhandari et al. [25], the mode of action of essential oils is not fully understood. Still, their effects on post-harvest phytopathogens are mainly attributed to their direct impact on spore germination and mycelial growth, disrupting cellular metabolism.

Previous studies have demonstrated the potential of essential oils as antimicrobial agents, likely due to their main volatile components or combinations [25,26,27,28,29]. Citrus peels can extract essential oils, with yields ranging from 0.2% to 1.0% depending on variety, agronomic conditions, and extraction methods [29,30]. Additionally, orange peel essential oil mainly consists of limonene, β-pinene, and myrcene [31].

Studies have shown that the volatile compounds in essential oils can cause cell membrane disruption, cytoplasmic disorganization, and inhibition of fungal reproduction, which are mechanisms that control *G. citri-aurantii* [5,32]. Cai et al. [18] reported that essential oils compounds such as D-limonene, citral, and eugenol disrupt fungal cell membranes and induce reactive oxygen species (ROS) formation, leading to cell death. These findings highlight the potential of essential oils for citrus citrus’s post-harvest treatment.

The present research demonstrated that Tahiti acid lime fruits treated curatively were more effective in controlling sour rot, reducing lesion diameter by 99.3% and achieving 96% control efficacy. Conversely, Serna-Escolano et al. [33] found that the incidence and severity of sour rot in treated mature lemons were significantly higher in curative experiments than in preventive ones, with values of 58.67%, 40.33%, and 62.33% for 25, 50 mM HP-β-CD-thymol, and propiconazole, respectively. Also, Regnier et al. [19] demonstrated that essential oils from *C. citratus*, *C. martinii*, *Origanum vulgare*, and *Geranium graveolens roseum* Bourbon (1000 µL L^−1^), incorporated into coatings or applied as curative dips, resulted in a 90% reduction in sour rot compared to the negative control.

Mandarin essential oils, such as those studied by Devite et al. [34], show great potential as alternatives to chemical fungicides for controlling phytopathogens. In their study, different concentrations of essential oils extracted from mandarin varieties effectively inhibited the mycelial growth of *Alternaria alternata*, a fungus that causes brown spots in citrus. The authors observed that essential oil from the IAC 2019Maria variety, applied at 16 µL mL^−1^, demonstrated the highest inhibition of fungal growth, significantly reducing disease severity in curative and preventive treatments.

The large-scale applicability of these essential oils has shown promise, mainly due to their biodegradability and low toxicity. However, challenges remain concerning their volatility and the need for microencapsulation to ensure controlled and prolonged release during storage [33,35,36]. Additionally, studies have shown that fruits treated with different essential oils maintain their quality parameters, underscoring the importance of understanding optimal concentrations and the antagonistic effects against post-harvest pathogens to enhance fruit quality and shelf life [37]. Future research should focus on optimizing formulations and exploring combinations of these oils to maximize their efficacy in controlling citrus pathogens. However, further research is required to fully elucidate the mechanisms of action and optimize the application methods, including microencapsulation, to enhance efficacy and longevity during storage. Adopting essential oils in commercial citrus operations could reduce reliance on chemical fungicides, addressing fungicide resistance issues and environmental pollution.

## 4. Material and Methods

### 4.1. Microorganism

This study was conducted using the isolate *G. citri-aurantii*, registered in SISGEN under number A03215C and belonging to the Microorganism Collection of the Phytopathology and Biological Control Laboratory at the Sylvio Moreira Advanced Citrus Research Center/IAC, Cordeirópolis. Pure and active colonies of the isolate were maintained on Petri plates containing Potato Dextrose Agar (PDA) medium in a biochemical oxygen demand (BOD) incubator at 27 °C for 7 days, with a 12-h photoperiod.

In in vitro assays, 5 mm fungal mycelium disks were used as inocula. For in vivo assays, the fungal mycelium was removed from the surface of Petri plates containing PDA medium and transferred to tubes containing autoclaved distilled water. The suspension was filtered through two layers of gauze, and the final inoculum suspension was adjusted to 10^4^ conidia mL^−1^, using a Neubauer chamber.

### 4.2. Essential Oils

The EO extraction process was carried out in the Laboratory of Analysis of Fruit Quality (LMQF) of the Sylvio Moreira Citrus Center (IAC). The essential oils were extracted by hydrodistillation using a modified Clevenger apparatus, an extractor apparatus idealized to obtain essential oils with the characteristics that make them valuable oils with high density as much as lower density than the water. Small amounts of oil can also be easily readable on the scale. It is compact and sturdy equipment that enables lower heating on vegetable material and makes convenient essential oil condensation possible [38]. For oil extraction, a 400 g sample of peel cut with approximately 1 cm^2^ of fresh fruit from 20 fruits was used with 800 mL of distilled water and heated to 100 °C on a heating mantle until the mixture reached boiling. The vapor and volatile compounds were directed to the condenser for heat exchange, condensing the vapors with cooling water. At this stage, the liquid forms of essential oil and water were visualized in the separator tube of the extractor, maintaining a continuous four-hour extraction cycle [38]. The oil was stored in an amber vial, protected from light, and kept at 4 °C.

Five essential oils were used in the experiments, from the following varieties: Late IAC 585, BRS Rainha, and Rio IAC 194 willowleaf mandarins (*Citrus* × *deliciosa*), Murcott IAC 221 tangor (*C.* × *sinensis* × *C. reticulata*), and Pera IAC sweet orange (*Citrus* × *sinensis*). Ripe fruit peels were used for the oil extraction, except for Late IAC 585 mandarin and Pera sweet orange, where the extraction was performed using green fruit peels.

The essential oil yield, expressed as a percentage, was also calculated on a wet basis (BU) through the following equation [38], as follows:

(1) $$\mathrm{Yield}\;(\%)=\frac{Volume\;of\;oil\;obtained\;(\mathrm{mL})}{Sample\;mass\;(g)}\times 100$$

### 4.3. Chemical Composition of EOs by GC-MS

Gas chromatography-mass spectrometry (GC–MS) analyses were carried out using a gas chromatograph Agilent 6890N with a mass spectrometer detector 5975MSD equipped with a fused silica capillary column HP5-MS (30 m × 0.25 mm × 0.25 μm). The oven temperature was programmed from 60° to 240 °C at 3 °C min^−1^. The carrier gas was He (1 mL min^−1^). The temperatures of both the injector and detector were 220 °C and 250 °C; 1 μL injection volume (sample 30 mg mL^−1^ in ethyl acetate); and split ratio 40:1. The compounds were identified from their retention indexes (RIs) on the database of the NIST 11 library, using a homologous series of n-alkanes (C8–C24) and by comparison of the MS data presented in the literature [39].

### 4.4. Bioactivity of Essential Oils on Mycelial Growth of Geotrichum citri-aurantii

(a) Antifungal Activity of Essential Oils by Direct Contact

Essential oils were incorporated into molten Potato Dextrose Agar—PDA medium (50 °C), supplemented with Tween 80 (0.5% *v*/*v*) and poured into 90 mm Petri dishes. The tested oil concentrations were 0 (control), 2, 4, 8, 16, and 32 µL mL^−1^. After 24 h, a 5 mm disc from the edge of a 7-day-old pathogen colony was placed in the center of each Petri dish, ensuring the fungal structures were in contact with the culture medium. The plates were incubated in a BOD chamber at 27 °C with a 12-h light/dark photoperiod for seven days.

The evaluation was performed by measuring the fungal colony diameter (two orthogonal measurements) daily until one of the treatments reached the total plate diameter. The values obtained were used to calculate the percentage inhibition of mycelial growth (PIMG) compared with the 0 µL mL^−1^ (control), the mycelial growth rate index through the sum of the evaluation interval (MGRI), and the area under the mycelial growth curve (AUMGC) equation proposed by Shaner and Finney [40], as shown in the equations below:

(2) $$\mathrm{PIMG}=\frac{\left(Controlgrowth-treatmentgrowth\right)}{Controlgrowth}\times 100$$

(3) $$\mathrm{MGRI}=\sum \frac{\left(Current\;average\;diameter-Previous\;average\;diameter\right)}{Number\;of\;days\;after\;inoculation}\;$$

(4) $$\mathrm{AUMGC}=\sum [(\frac{\left(Yi+Yi+1\right)}{2})\times (Ti+1-Ti)],$$

where *Yi* and *Yi* + 1 are the colony growth values observed in two consecutive evaluations, and (Ti + 1 − Ti) is the time interval between evaluations.

(b) Antifungal Activity of Essential Oil Volatile Compounds

The concentrations of 0 (control), 2, 4, 8, 16, and 32 µL mL^−1^ of each essential oil were tested to assess the effect of volatile compounds. The essential oil solution was prepared by adding Tween 80 (0.5% *v*/*v*) and distilled water. A 20 µL aliquot of each concentration was deposited onto a sterile filter paper disc (5 mm), which was then placed in one compartment of a two-compartment Petri dish (90 × 15 mm). In the opposite compartment, a 5 mm disc of pathogen mycelium was placed on the PDA with the mycelium facing upward. The plates were sealed with PVC film and incubated at 27 °C with a 12-h light/dark photoperiod for four days. The colony diameter was measured daily. The values obtained were used to calculate the percentage inhibition of mycelial growth relative to the 0 µL mL^−1^ (control), the mycelial growth rate index, and the area under the mycelial growth curve (AUMGC), as described in the previous section.

### 4.5. Effectiveness of Essential Oils in Controlling Sour Rot in Post-Harvest Citrus

Tahiti acid lime fruits were collected, sanitized with neutral detergent, and surface disinfected with 0.1% sodium hypochlorite for two minutes. After drying, the fruits were wounded at two equidistant points on the equatorial region of the fruits using sterilized needles to a depth of 3 mm. The fruits were then inoculated at the wound site with 20 µL of a *G. citri-aurantii* spore suspension containing a concentration of 1 × 10^4^ conidia mL^−1^ 24 h after (preventive treatment) and 24 h before (curative treatment) the application of essential oil from Late willowleaf or Pera IAC sweet orange, at doses of 32 and 64 µL mL^−1^, selected through in vitro assays. The essential oil solution was prepared by adding Tween 80 (0.5% *v*/*v*) and distilled water. In the control treatment, the fruits were treated only with water. The fruits were stored under ambient conditions (25 °C ± 2 and 70% relative humidity).

The severity of the disease was evaluated daily by measuring the diameter of the lesions formed. The obtained measurements were used to determine the area under the disease progress curve (AUDPC) using the equation proposed by Shaner and Finney [40]:

(5) $$\mathrm{AUDPC}=Ʃ[\frac{\left(Yi+Yi+1\right)}{2}\times (Ti+1-Ti)]$$

where *Yi* is the lesion diameter at time *Ti* (in days) and *Yi* + 1 is the lesion diameter at time *Ti* + 1.

The percentage of healthy fruits determined the incidence. The experimental design for the severity and incidence measurements was completely randomized in a triple factorial scheme with additional treatment (2 × 2 × 2 + 1), where factor 1 refers to the fruit treatment (preventive and curative), factor 2 to the essential oils evaluated (Late IAC 585 willowleaf mandarin and Pera IAC sweet orange), and factor 3 to the doses (32 and 64 µL mL^−1^), with the additional treatment being the control. Each treatment consisted of 3 replications, with 10 fruits per replication.

### 4.6. Fruit Quality in Citrus After Oleo Essential Treatment

The fruits were preprocessed as described in Section 2.3. Tahitian sour lime fruits were spray-treated with different concentrations (0 (control), 32, and 64 µL mL^−1^) of Willowleaf Late mandarin IAC 585 and sweet orange IAC Pera essential oils. The quality parameters evaluated were average juice yield, total soluble solids (SS), total titratable acidity (TTA), and the SS/TTA ratio. The percentage juice yield was calculated using % juice yield = (MS/MF) × 100, where MS is the juice mass and MF is the fruit mass. The total soluble solids (SS) content was measured using a digital refractometer, with results expressed in °Brix. Total titratable acidity (TTA) was determined using an automatic titrator, and the results were expressed as % (g citric acid per 100 mL sample). The SS to TTA ratio was calculated by dividing the SS content by the TTA. The average values were determined from 10 fruits per treatment 24 h after treatment, followed by evaluations after 7 and 14 days of storage at ambient conditions (25 °C ± 2 and 70% relative humidity).

### 4.7. Statistical Analyses

The data obtained were initially subjected to analysis of variance (ANOVA), and subsequently, the means were compared using Tukey’s test at a 5% significance level using the R software, version 4.4.1 for Windows [41]. Data normality was verified using the Shapiro–Wilk test at a 5% significance level. Principal Component Analysis (PCA) was also applied to the in vitro essay [41] to al-low a global analysis of the results . The Bip-lot contain all 39 volatile compounds of the essential oils used (Late IAC 585, BRS Rainha, Rio IAC 194 willowleaf mandarins, Murcott IAC 221 tangor and Pera IAC sweet orange) and MGRI, AUMGC, and PIMG values at a dose 32 µL mL.

## 5. Conclusions

The study demonstrated that Pera IAC sweet orange and Late IAC 585 willow leaf essential oils offer promising potential as natural antifungal agents for controlling postharvest citrus sour rot, specifically Tahiti lime fruit. Pera IAC sweet orange essential oil, at a concentration of 32 µL mL^−1^, effectively reduced disease severity by approximately 96% in curative treatments, with no apparent adverse effects on fruit quality. The research also highlighted the importance of volatile compounds present in essential oils, which act by inhibiting the mycelial growth of the fungus *Geotrichum citri-aurantii* and their potential antifungal properties. These results suggest that citrus essential oils may be a viable alternative to synthetic fungicides; however, it is essential to continue studies on the large-scale application of these oils, exploring the encapsulation of volatile compounds to ensure a controlled and prolonged release during storage. These innovations could benefit the citrus sector by promoting safer production with fewer chemical residues, meeting the demands of consumers and exporters for sustainable practices with low environmental impact.

## Figures and Tables

**Table 1:** Peel essential oil chemical composition of different citrus evaluated (IAC, Cordeirópolis-SP, Brazil).

Volatile Compounds	RI_calc_^1^	Pera IAC	Late IAC 585	Rio IAC 194	BRS Rainha	Murcott IAC 221
		Relative Percentage (%)				
2-Thujene	926	0.36	0.48	0.49	0.52	0.00
α-Pinene	933	1.09	1.91	1.41	1.41	0.48
Sabinene	973	0.27	0.40	0.27	0.27	0.41
β-Pinene	977	1.28	0.91	1.14	1.56	0.06
β-Mircene	991	2.01	2.59	2.12	1.81	2.00
Octanal	1003	0.15	0.00	0.16	0.15	2.30
α-Phellandrene	1006	0.09	0.84	0.07	0.07	0.00
α-Terpinene	1017	0.49	0.30	0.32	0.46	0.00
o-Cymene	1025	0.65	0.92	2.51	3.10	0.00
Limonene	1032	66.81	76.53	71.24	62.40	81.92
trans-β-Ocimene	1047	0.00	0.16	0.00	0.00	0.00
γ-Terpinene	1059	17.59	10.64	15.86	17.67	0.12
1-Octanol	1070	0.00	0.00	0.00	0.00	1.92
Terpinolene	1088	1.06	0.61	0.86	1.02	0.00
Linalool	1101	0.74	0.96	0.45	1.08	5.80
Nonanal	1105	0.00	0.00	0.00	0.00	0.39
Citronellal	1152	0.08	0.00	0.13	0.12	0.65
1-Nonanol	1171	0.00	0.00	0.00	0.00	0.10
Terpinen-4-ol	1177	0.83	0.12	0.17	1.05	0.20
α-Terpineol	1191	1.68	0.16	0.31	1.77	0.55
Decanal	1205	0.14	0.00	0.16	0.13	1.11
cis-Carveol	1219	0.00	0.00	0.00	0.08	0.00
Citronellol	1229	0.37	0.00	0.24	0.56	1.19
Carvone	1243	0.00	0.00	0.00	0.09	0.10
Geranial	1270	0.00	0.00	0.00	0.12	0.00
Perillaldehyde	1273	0.42	0.00	0.17	0.46	0.46
Limone-10-ol	1290	0.12	0.00	0.00	0.18	0.15
Thymol	1296	0.19	0.11	0.10	0.34	0.00
Neryl acetate	1365	0.00	0.00	0.00	0.00	0.10
Methyl N-methylanthranilate	1406	2.86	0.00	1.24	2.86	0.00
Caryophyllene	1418	0.13	0.22	0.00	0.10	0.00
β-Farnesene	1457	0.00	0.50	0.00	0.00	0.00
Germacrene D	1479	0.00	0.17	0.00	0.00	0.00
Bicyclogermacrene	1495	0.00	0.90	0.00	0.00	0.00
α-Farnesene	1508	0.19	0.00	0.31	0.25	0.00
δ-Cadinene	1522	0.00	0.17	0.00	0.00	0.00
NI ^2^	1555	0.00	0.19	0.00	0.00	0.00
Spathulenol	1576	0.00	0.21	0.00	0.00	0.00
α-Sinesal	1753	0.41	0.00	0.30	0.37	0.00

^1^ RIcalc—Retention Index calculated. ^2^ NI—Not identified.

**Table 2:** Effect of essential oil doses on antifungal activity by contact: mycelial growth rate index (MGRI), the area under the mycelial growth curve (AUMGC), and percentage mycelial growth inhibition (PIMG) of *G. citri-aurantii* compared to the control (cm) in vitro.

Essential Oil Samples	Doses (µL mL^−1^)	MGRI	AUMGC	PIMG
BRS Rainha willowleaf	2	1.41 bc *	25.13 bc	2.4 ab
	4	1.67 ab	27.50 ab	1.2 ab
	8	1.57 abc	25.38 bc	6.2 ab
	16	1.63 ab	25.94 bc	2.5 ab
	32	1.76 a	22.77 bc	12.0 a
	Tween	1.37 c	24.49 bc	10.6 a
	Control	1.64 ab	30.75 a	
	CV (%) **	7.2	7.1	
Murcott IAC 221 tangor	2	1.81 a	27.69 ab	0.70 a
	4	1.52 b	21.83 c	1.64 a
	8	1.19 c	17.92 d	1.87 a
	16	1.25 c	18.64 d	2.25 a
	32	-	-	-
	Tween	1.64 ab	27.08 b	0.70 a
	Control	1.64 ab	30.75 a	
	CV (%)	5.88	5.91	
Rio IAC 194 Willowleaf	2	1.82 c	23.61 bc	12.5 a
	4	1.92 bc	24.83 bc	8.48 ab
	8	1.83 bc	23.30 bc	8.95 ab
	16	1.81 c	22.99 c	10.63 ab
	32	1.92 bc	24.80 bc	5.13 ab
	Tween	2.06 ab	25.54 b	3.53 b
	Control	2.29 a	29.39 a	
	CV (%)	6.1	4.64	
Late IAC 585 willowleaf	2	1.71 ab	23.18 a	0.63 c
	4	2.04 a	26.30 a	4.5 abc
	8	1.95 a	24.89 a	7.75 bc
	16	2.12 a	28.33 a	11.0 b
	32	0.99 b	14.00 b	44.38 a
	Tween	2.06 a	25.55 ab	2.12 bc
	Control	2.29 a	29.38 a	
	CV (%)	21.46	20.89	
Pera IAC	2	1.72 a	22.09 b	0 b
	4	1.56 ab	30.38 b	0.73 b
	8	1.71 a	19.28 b	0 b
	16	1.23 b	13.43 c	23.70 a
	32	1.27 b	11.51 c	24.83 a
	Tween	1.64 ab	21.53 b	0 b
	Control	1.83 a	25.66 a	
	CV (%)	8.70	5.79	

* The means in columns for each essential oil followed by the same letter do not differ according to the Tukey test at a significance level of 5%; ** Coefficient of variation.

**Table 3:** Effect of essential oil doses on the antifungal activity of volatile compounds: mycelial growth rate index (MGRI), the area under the mycelial growth curve (AUMGC), and percentage mycelial growth inhibition (PIMG) of *G. citri-aurantii* compared to the control (cm) in vitro.

Essential Oil Samples	Doses (µL mL^−1^)	MGRI	AUMGC	PIMG
BRS Rainha willowleaf	2	1.69 b *	8.02 c	28.22 a
	4	1.76 b	8.46 bc	24.48 a
	8	1.73 b	8.39 bc	25.31 a
	16	1.78 b	8.52 bc	23.23 a
	32	1.94 ab	7.69 c	27.38 a
	Tween	1.62 b	9.00 b	24.06 a
	Control	2.2 a	11.27 a	-
	CV (%) **	9.97	4.54	17.69
Murcott IAC 221 tangor	2	1.69 a	8.20 c	16.75 a
	4	1.76 a	8.46 bc	12.92 ab
	8	1.73 a	8.39 c	13.87 ab
	16	1.78 a	8.52 bc	11.48 ab
	32	-	-	-
	Tween	1.25 a	9.54 b	5.26 b
	Control	1.73 a	11.17 a	-
	CV (%)	15.08	5.60	34.80
Rio IAC 194 Willowleaf	2	2.03 a	8.37 a	4.08 a
	4	1.95 a	7.79 a	6.24 a
	8	1.95 a	8.19 a	6.02 a
	16	1.90 a	7.69 a	7.09 a
	32	1.92 a	7.86 a	11.61 a
	Tween	1.87 a	7.95 a	9.46 a
	Control	1.80 a	8.42 a	-
	CV (%)	7.86	8.33	32.62
Late IAC 585 willowleaf	2	1.67 ab	9.32 b	2.72 b
	4	1.83 a	9.35 b	5.44 b
	8	1.87 a	9.68 ab	6.46 b
	16	1.50 b	6.25 c	29.93 a
	32	1.49 b	5.84 c	30.95 a
	Tween	1.60 ab	8.74 b	6.42 b
	Control	1.65 ab	10.36 a	-
	CV (%)	6.01	4.16	18.79
Pera IAC	2	1.67 ab	8.82 a	2.72 b
	4	1.85 a	8.45 a	4.76 b
	8	1.85 a	8.16 a	7.14 b
	16	1.50 b	5.96 b	29.93 a
	32	1.49 b	5.64 b	30.95 a
	Tween	1.60 ab	8.76 a	4.76 b
	Control	1.66 ab	9.04 a	-
	CV (%)	5.77	6.08	16.88

* The means in columns for each essential oil followed by the same letter do not differ according to the Tukey test at a significance level of 5%; ** Coefficient of variation.

**Table 4:** The area under the disease progress curve (AUDPC), evaluated by the average lesion diameter (mm) caused by *G. citri-aurantii* in Tahiti acid lime fruits treated with preventively and curatively with concentrations of 32 and 64 µL mL^−1^.

Treatments	Doses	
	32 µL mL^−1^	64 µL mL^−1^
Preventive	62.31 Aa *	53.47 Aa
Curative	17.30 Bb	61.17 Aa

* Means followed by the same letter in the row or column do not differ at a 5% significance level. Tukey’s test applied to columns (uppercase letters) and rows (lowercase letters).

**Table 5:** The area under the disease progress curve (AUDPC), evaluated by the average lesion diameter (mm) caused by *G. citri-aurantii* in Tahiti acid lime fruits treated with Late willowleaf mandarin and Pera IAC sweet orange essential oils at concentrations of 32 and 64 µL mL^−1^.

Essential Oils	Doses	
	32 µL mL^−1^	64 µL mL^−1^
Late	56.02 Aa *	45.10 Aa
Pera IAC	23.59 Bb	69.54 Aa

* Means followed by the same letter in the row or column do not differ at a 5% significance level. Tukey’s test applied to columns (uppercase letters) and rows (lowercase letters).

**Table 6:** Disease incidence was evaluated by the percentage of fruits showing sour rot symptoms caused by *G. citri-aurantii* in Tahiti acid lime fruits treated preventively and curatively with 32 and 64 µL mL^−1^ of Late willowleaf mandarin and Pera IAC sweet orange essential oils.

Treatments	Doses	
	32 µL mL^−1^	64 µL mL^−1^
Preventive	72.90 Aa *	63.89 Ba
Curative	32.22 Bb	91.48 Aa

* Means followed by the same letter in the row or column do not differ at a 5% significance level. Tukey’s test applied to columns (uppercase letters) and rows (lowercase letters).

**Table 7:** Disease incidence was evaluated by the percentage of fruits showing sour rot symptoms caused by *Geotrichum citri-aurantii* in Tahiti acid lime fruits treated with Late willowleaf mandarin and Pera IAC sweet orange essential oils at concentrations of 32 and 64 µL mL^−1^.

Essential Oils	Doses	
	32 µL mL^−1^	64 µL mL^−1^
Late	69.89 Aa *	70.14 Aa
Pera IAC	35.23 Bb	85.23 Aa

* Means followed by the same letter in the row or column do not differ at a 5% significance level. Tukey’s test applied to columns (uppercase letters) and rows (lowercase letters).

**Table 8:** Physicochemical characteristics of Tahiti acid lime fruits treated with Late willowleaf mandarin and Pera IAC sweet orange essential oils at concentrations of 32 and 64 µL mL^−1^ and stored at 25 °C ± 2, 2023 harvest (Citrus Center Silvio Moreira—Cordeirópolis, SP).

Juice Yield			
Treatments	One Day	7 Days	14 Days
**Control**	44.4 *	48.8	51.6
Late (32 µL mL^−1^)	52.6	51.5	51.9
Late (64 µL mL^−1^)	52.0	53.6	50.0
Pera IAC (32 µL mL^−1^)	50.3	44.2	50.3
Pera IAC (64 µL mL^−1^)	49.4	45.9	54.6
**Acidity (g 100 mL^−1^)**			
**Treatments**	**1 Day**	**7 Days**	**14 Days**
Control	5.98	5.96	5.28
Late (32 µL mL^−1^)	5.83	6.00	6.27
Late (64 µL mL^−1^)	6.78	4.14	5.19
Pera IAC (32 µL mL^−1^)	5.92	5.98	6.36
Pera IAC (64 µL mL^−1^)	5.81	5.28	5.67
**Total Soluble Solids (°Brix)**			
**Treatments**	**1 Day**	**7 Days**	**14 Days**
Control	8.9	9.2	10.1
Late (32 µL mL^−1^)	9.3	9.7	9.8
Late (64 µL mL^−1^)	6.78	9.1	9.5
Pera IAC (32 µL mL^−1^)	5.92	9.4	9.9
Pera IAC (64 µL mL^−1^)	5.81	9.2	9.5
**Ratio**			
**Treatments**	**1 Day**	**7 Days**	**14 Days**
Control	1.5	1.5	1.9
Late (32 µL mL^−1^)	1.6	1.6	1.6
Late (64 µL mL^−1^)	1.4	2.2	1.8
Pera IAC (32 µL mL^−1^)	1.6	1.6	1.6
Pera IAC (64 µL mL^−1^)	1.5	1.7	1.7

* Mean values of samples containing ten fruits.

## Data Availability

Data are contained within the article.
